# Supplementary material for: Comparison of Methods for Incorporating Related Data When Developing Clinical Prediction Models: A Simulation Study
Source: Biom J. 2026 Aug 27;68(5):e70166. doi: 10.1002/bimj.70166 (PMC13520826; doi:10.1002/bimj.70166)
Supplement: Supplementary file 2 [file BIMJ-68-e70166-s001.zip › third submission code review - R2/reproducibility_supplement/results/Plots/combined_plots/sampsize_plot_combined_SC_ID1_TS0.25_model15.pdf]

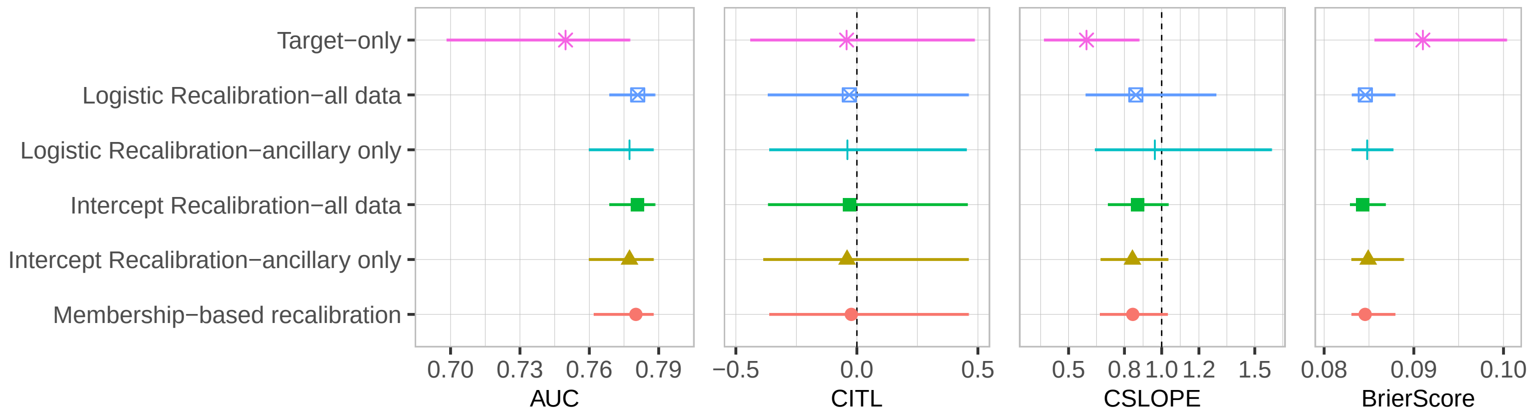

(a) (N_{min})

source and target insufficient

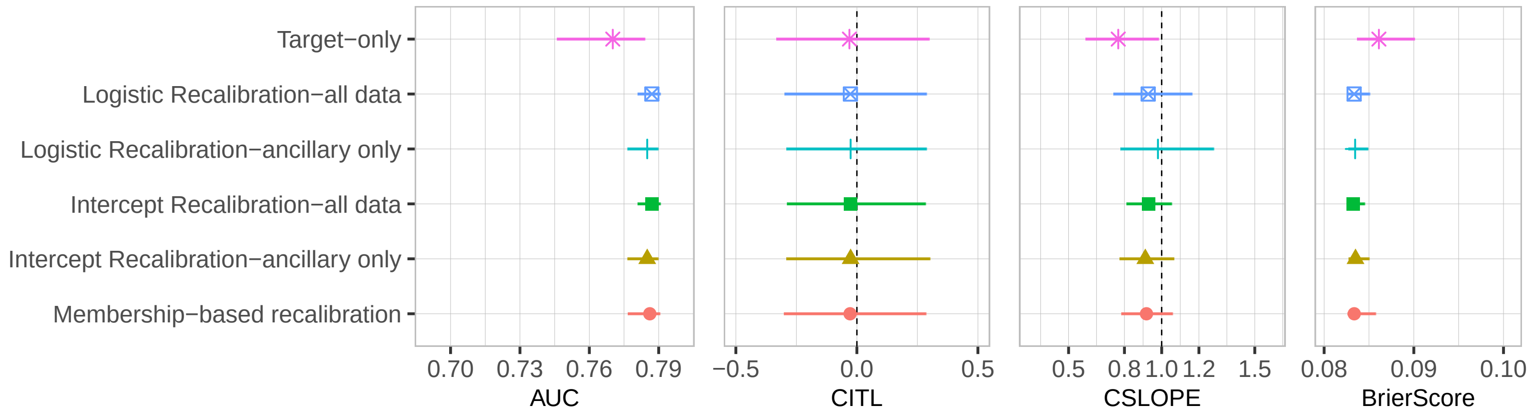

(b) ($2 \times N_{min}$)

source sufficient / target insufficient

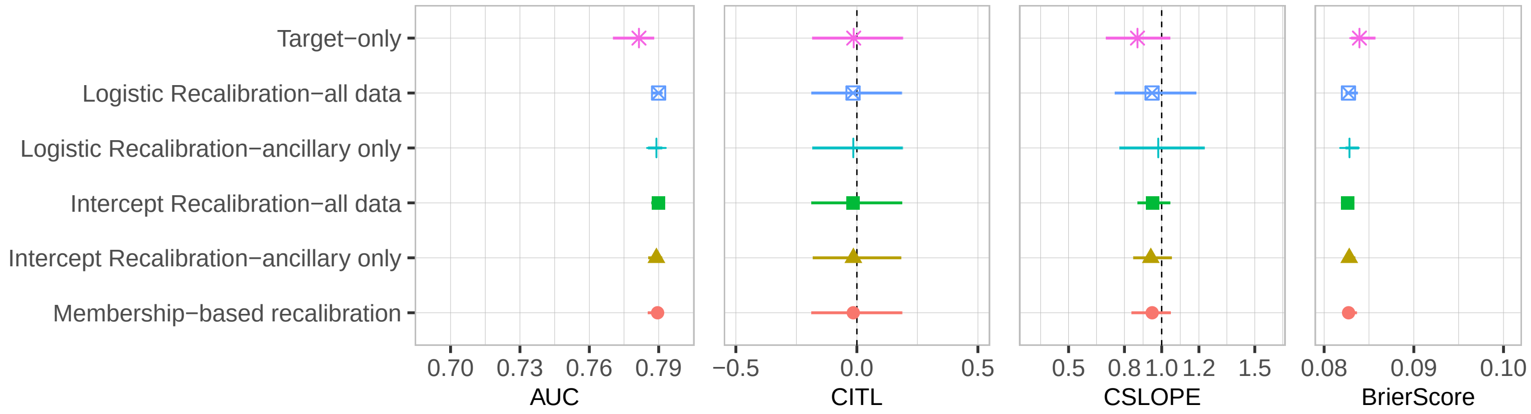

(c) ($4 \times N_{min}$)

source and target sufficient
